# Late-Onset Prosthetic Valve Dysfunction Associated With Hypoattenuated Leaflet Thickening Detected After a New Heart Murmur: A Case Report

**DOI:** 10.7759/cureus.116665

**Published:** 2026-09-21

**Authors:** Kazuyoshi Takagi, Kosuke Saku, Hiroki Mine, Takanori Kono, Eiki Tayama

**Affiliations:** 1 Division of Cardiovascular Surgery, Department of Surgery, Kurume University, Kurume, JPN

**Keywords:** anticoagulation therapy, hypo-attenuated leaflet thickening, imaging evaluation, prosthetic valve dysfunction, sutureless bioprosthetic valve

## Abstract

Hypoattenuated leaflet thickening (HALT) is increasingly recognized after bioprosthetic aortic valve replacement; however, its clinical significance and optimal detection strategy in routine practice remain uncertain. We report the case of a 79-year-old woman who underwent minimally invasive aortic valve replacement using a Perceval S sutureless bioprosthesis for severe aortic stenosis. Early postoperative transthoracic echocardiography and cardiac computed tomography demonstrated normal prosthetic valve function without evidence of HALT. Oral anticoagulation was discontinued three months after surgery, and the patient remained asymptomatic. At the one-year follow-up visit, a newly detected systolic heart murmur prompted further evaluation despite stable N-terminal pro-B-type natriuretic peptide levels. Echocardiography revealed increased transvalvular gradients and mild-to-moderate transvalvular regurgitation, while cardiac computed tomography demonstrated HALT involving the right and noncoronary cusps. The combination of hemodynamically significant prosthetic valve dysfunction and CT-confirmed HALT was considered consistent with clinically overt bioprosthetic valve thrombosis. Oral anticoagulation was restarted, followed by complete resolution of HALT and improvement in prosthetic valve hemodynamics. This case demonstrates that clinically overt bioprosthetic valve thrombosis associated with HALT may develop in an asymptomatic patient and that a newly detected cardiac murmur may serve as a clinical trigger for further echocardiographic and CT evaluation. Careful routine clinical follow-up may facilitate recognition of hemodynamically relevant prosthetic valve dysfunction and appropriate therapeutic intervention.

## Introduction

Subclinical leaflet thrombosis, characterized by hypoattenuated leaflet thickening (HALT) on four-dimensional cardiac computed tomography (cCT), has been increasingly recognized after both transcatheter and surgical bioprosthetic aortic valve replacement [1-3]. HALT may occur without clinically apparent prosthetic valve dysfunction (PVD) but can be accompanied by reduced leaflet motion (RLM) and, in some patients, alterations in valve hemodynamics. The reported prevalence of HALT varies considerably according to prosthesis type, study population, timing of CT assessment, and imaging methodology. In the Evolut Low Risk CT substudy, HALT was observed at 30 days in 17.3% of patients after transcatheter aortic valve replacement (TAVR) and 16.5% after surgical aortic valve replacement (SAVR), increasing to 30.9% and 28.4%, respectively, at one year [2]. In the PARTNER 3 (Placement of Aortic Transcatheter Valves 3) CT substudy, HALT was identified in 13% of TAVR and 5% of SAVR patients at 30 days and in 28% and 20%, respectively, at one year [3]. Serial CT assessment in the PARTNER 3 substudy also demonstrated the dynamic nature of HALT: 54% of HALT detected at 30 days had resolved by one year, whereas new HALT developed in 21% of patients without HALT at 30 days [3]. These observations were derived from specific low-risk TAVR and SAVR populations using serial four-dimensional CT and should not be directly extrapolated to other bioprosthetic valve types.

Data specific to sutureless surgical bioprostheses are more limited. Dalén et al. evaluated 47 patients with a Perceval S sutureless bioprosthesis using cCT at a median of 491 days (range, 36-1247 days) after implantation and reported HALT in 38% and RLM in 28% of patients [4]. All patients with RLM also had HALT. Given the wide range in timing of CT assessment and differences in prosthesis design and imaging protocols across studies, the prevalence and natural history of HALT specifically after Perceval S implantation remain incompletely defined.

Although the diagnosis and treatment of clinically overt bioprosthetic valve thrombosis (BPVT) have been described [5,6], the clinical significance of HALT and its relationship with hemodynamically significant PVD remain incompletely understood [1-6]. This case illustrates the clinical distinction between incidental HALT and HALT associated with hemodynamically significant PVD and demonstrates how a subtle change in physical examination prompted further multimodality evaluation.

## Case presentation

A 79-year-old woman with a history of heart failure presented with very severe aortic stenosis (peak velocity (PV) 5.6 m/s, peak pressure gradient (PPG) 125 mmHg, and aortic valve area 0.7 cm²). She underwent minimally invasive aortic valve replacement (AVR) with a Perceval S sutureless bioprosthesis (PerSV), size S. Continuous intravenous heparin was initiated on postoperative day (POD) 1, targeting an activated partial thromboplastin time of 45-55 seconds. Oral vitamin K antagonist (VKA) therapy was subsequently started with a target prothrombin time-international normalized ratio (PT-INR) of 1.8-2.2, in combination with low-dose aspirin (100 mg).

cCT was performed using a Revolution CT scanner (GE Healthcare, Chicago, Illinois) with ECG-gated image acquisition. Images were reconstructed at a slice thickness of 0.625 mm, and approximately 1 mL/kg of iodinated contrast medium was administered. Heart rate control was performed when necessary according to standard institutional practice, with beta-blockers administered when deemed appropriate to optimize image quality during ECG-gated acquisition. HALT was assessed on multiplanar reconstructed images, including long-axis images aligned with the center of each prosthetic leaflet. Leaflet motion was also assessed on ECG-gated images for the presence of RLM.

TTE performed on POD 7 showed normal prosthetic valve function (Figure 1a, Table 1), and ECG-gated cCT demonstrated no evidence of HALT or RLM (Figure 1b,c). Physical examination revealed no cardiac murmur. The patient was discharged on VKA and aspirin without complications.

At three months after surgery, TTE continued to show normal prosthetic valve function, and N-terminal pro-B-type natriuretic peptide (NT-proBNP) levels showed gradual improvement over the postoperative course (Table 1). VKA was then discontinued, and aspirin alone was maintained. At 12 months postoperatively, the patient remained asymptomatic, but a newly developed grade 3/6 systolic ejection murmur was detected at the right second intercostal space along the sternal border. Although NT-proBNP levels showed no significant change (249.5 pg/mL at three months and 271.6 pg/mL at 12 months), TTE demonstrated new hemodynamic PVD. Compared with the three-month examination, PV increased from 2.7 to 3.7 m/s, PPG from 29.2 to 54.8 mmHg, and mean pressure gradient (MPG) from 14 to 28 mmHg, while the Doppler velocity index (DVI) decreased from 0.54 to 0.38. In addition, new mild-to-moderate transvalvular regurgitation (TVL) was observed (Figure 1d, Table 1). ECG-gated cCT demonstrated HALT predominantly involving the right and noncoronary cusps, accompanied by RLM (Figure 1e,f, Table 1). These findings indicated hemodynamically significant PVD associated with HALT and RLM rather than isolated or subclinical HALT.

The diagnosis of clinically overt BPVT was based not on the presence of HALT alone but on the combination of newly developed hemodynamic PVD and CT-confirmed HALT with associated RLM. Prosthetic valve endocarditis was considered unlikely because the patient had no fever or laboratory evidence of systemic inflammation, and TTE showed no vegetation. Although pannus formation could not be definitively excluded based on imaging findings at the 12-month evaluation, leaflet thrombosis was considered the most likely cause of the PVD based on the overall clinical, echocardiographic, and CT findings. These hemodynamic changes met the Valve Academic Research Consortium 3 (VARC-3) criteria for stage 2 moderate hemodynamic valve deterioration, based on an increase in MPG from 14 to 28 mmHg and a decrease in DVI from 0.54 to 0.38 compared with the three-month assessment [6]. Accordingly, the patient was diagnosed with clinically overt BPVT, and VKA therapy was reinitiated with a target PT-INR of 2.0-2.5.

After four months of resumed anticoagulation therapy, TTE demonstrated complete resolution of the TVL (Figure 1g) and improvement in prosthetic valve hemodynamics: PV decreased from 3.7 to 3.2 m/s, PPG from 54.8 to 41.0 mmHg, and MPG from 28 to 22 mmHg, while DVI increased from 0.38 to 0.45 (Table 1). ECG-gated cCT confirmed complete resolution of the previously observed HALT and associated RLM (Figure 1h,i). The concordant resolution of HALT and RLM and improvement in prosthetic valve hemodynamics following anticoagulation strongly supported leaflet thrombosis, rather than pannus formation or structural valve degeneration, as the underlying mechanism of PVD, although a causal therapeutic effect of anticoagulation cannot be established from this single case. No clinical evidence of thromboembolism occurred throughout the course. As the patient declined continuation of VKA after resolution of PVD, VKA was discontinued, and aspirin alone was maintained. No recurrence of PVD was observed up to two years postoperatively (Table 1). 

**Figure 1:**
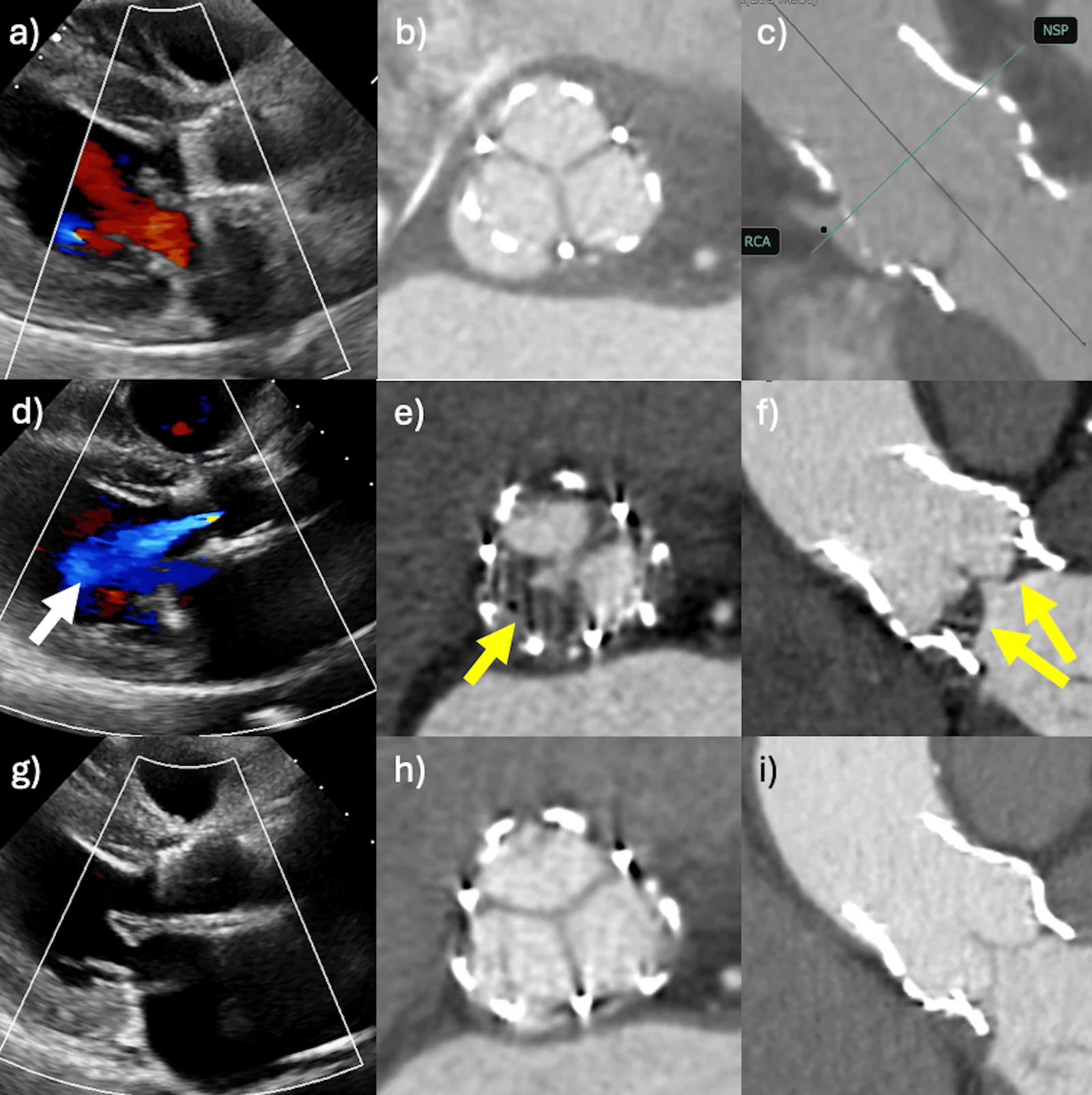
Serial echocardiographic and cardiac CT findings after Perceval S implantation. (a–c) Early postoperative assessment (POD 7): TTE showed normal prosthetic valve function (a), and ECG-gated cardiac CT with multiplanar reconstruction showed no evidence of HALT or RLM (b, c). (d–f) Twelve-month assessment: TTE demonstrated prosthetic valve dysfunction with newly developed TVL (white arrow) (d). ECG-gated cardiac CT demonstrated HALT predominantly involving the right and non-coronary cusps, accompanied by RLM (yellow arrows) (e, f). (g–i) After four months of resumed VKA therapy: TTE demonstrated resolution of the TVL and improvement in prosthetic valve hemodynamics (g), while cardiac CT confirmed resolution of HALT and associated RLM (h, i). CT images were reconstructed using multiplanar views, including long-axis images aligned with the center of the prosthetic leaflets. HALT, hypo-attenuated leaflet thickening; RLM, reduced leaflet motion; TTE, transthoracic echocardiography; CT, computed tomography; transvalvular regurgitation; TVL, VKA, vitamin K antagonist; POD, postoperative day.

**Table 1:** Serial changes in echocardiographic aortic valve findings, NT-pro BNP levels, and anticoagulation therapy. SAVR; surgical aortic valve replacement, TVL; transvalvular leakage, PVL; paravalvular leakage, PV; peak velocity, PPG; peak pressure gradient, MPG; mean pressure gradient, AVA; aortic valve area, AVAi: aortic valve area index, DVI; Doppler velocity index, NT-pro BNP; N-terminal prohormone of brain natriuretic peptide, PT-INR; prothrombin time-international normalized ratio VKA; oral Vitamin K antagonist, Asp; aspirin 100 mg.

Variables	Normal range	Preoperative	1 week after SAVR	3 months after SAVR	12 months after SAVR	16 months after SAVR	24 months after SAVR
TVL		-	none	none	mid-moderate	none	none
PVL		-	none	none	none	none	none
PV (m/s)		5.6	2.8	2.7	3.7	3.2	3.1
PPG (mmHg)		125.4	31.4	29.2	54.8	41.0	38.4
MPG (mmHg)		69.0	16.0	14.0	28.0	22.0	19.0
AVA (cm^2^)		0.70	1.36	1.08	0.84	1.14	1.14
AVAi (cm^2^/m^2^)		0.51	1.0	0.82	0.63	0.87	0.87
DVI	>0.3	0.20	0.40	0.54	0.38	0.45	0.49
NT pro BNP (pg/mL)	<125	698.4	390.7	249.5	271.6	244.0	237.8
PT-INR	0.8〜1.2	1.05	1.61	1.89	0.99	2.00	0.99
VKA		none	Yes	Yes	none	Yes	none
Asp		none	Yes	Yes	Yes	Yes	Yes

## Discussion

HALT after PerSV implantation has been reported to be relatively common, although its clinical significance remains uncertain [4]. In the study by Dalén et al., HALT was not clearly associated with significant hemodynamic deterioration or thromboembolic events, and no significant association was observed between ongoing anticoagulation therapy and the presence of HALT [4]. Importantly, these observations relate primarily to the association between anticoagulation and the development or presence of HALT and should be distinguished from the therapeutic effect of anticoagulation once HALT-associated PVD or clinically overt BPVT has developed. Other reports have described patients in whom echocardiographic evidence of PVD led to the identification of HALT, with subsequent improvement in leaflet abnormalities and valve hemodynamics following anticoagulation therapy [7,8]. In addition, late-onset BPVT after PerSV implantation has been reported in a symptomatic patient who required redo AVR and had extensive thrombus formation involving multiple leaflets [9]. These findings illustrate the heterogeneous clinical presentation of leaflet thrombosis after PerSV implantation, ranging from incidental HALT to hemodynamically significant valve dysfunction. Pathological studies of transcatheter bioprostheses have further supported the thrombotic nature of HALT, although these findings cannot necessarily be extrapolated directly to sutureless surgical bioprostheses [10].

HALT is increasingly recognized after bioprosthetic valve implantation [1-6]; however, incidental HALT without hemodynamic valve dysfunction should be distinguished from clinically significant BPVT. Routine therapeutic intervention for incidentally detected HALT is not currently recommended, and management should be individualized according to symptoms, valve hemodynamics, imaging findings, thromboembolic risk, and bleeding risk [5,6,11]. Routine surveillance with cCT is also not generally recommended [11]. Notably, the patient remained asymptomatic and showed no substantial change in NT-proBNP despite the development of hemodynamically significant PVD. In this patient, the newly detected systolic murmur served as the clinical finding that prompted echocardiographic evaluation, which identified new hemodynamically relevant PVD, followed by cCT demonstrating HALT with associated RLM. Cardiac auscultation therefore did not directly detect HALT or establish the diagnosis of BPVT. Rather, this case illustrates how a change in physical examination findings may prompt further evaluation of prosthetic valve function in an individual patient; it does not establish the diagnostic performance or clinical utility of auscultation as a screening strategy for HALT or BPVT.

The relationship between HALT and clinically overt BPVT remains incompletely understood [6]. HALT is an imaging finding that may occur without symptoms or hemodynamic deterioration and should not be considered synonymous with clinically overt BPVT. Previous studies have shown that increases in transvalvular gradients, leaflet thickening, and abnormal leaflet mobility are useful features for distinguishing BPVT from structural valve deterioration [12]. In the present case, the diagnosis of BPVT was supported by the coexistence of CT-confirmed HALT with RLM and newly developed hemodynamic PVD, including increased transvalvular gradients, decreased DVI, and transvalvular regurgitation. Prosthetic valve endocarditis was considered unlikely because of the absence of fever, systemic inflammatory findings, and vegetation on echocardiography. Although pannus formation could not be definitively excluded by imaging at the 12-month evaluation, the subsequent resolution of HALT and RLM together with improvement in valve hemodynamics following VKA therapy strongly supported a thrombotic mechanism.

Anticoagulation has been associated with improvement or resolution of established HALT and BPVT in previous reports and studies [7,8,13,14]. In the present case, HALT and RLM disappeared, and valve hemodynamics improved after four months of VKA therapy. Although the temporal and concordant anatomical and hemodynamic response supports thrombotic valve dysfunction as the underlying mechanism, a causal therapeutic effect of anticoagulation cannot be established from a single uncontrolled case. Spontaneous changes in HALT have been documented during longitudinal follow-up [2,3], further limiting causal inference from this observation alone.

This distinction is particularly important when considering patients without symptoms. The present patient was clinically asymptomatic but did not have isolated or incidental HALT; she had objective hemodynamic PVD associated with HALT and RLM. Therefore, the observed response to VKA should not be extrapolated to support routine anticoagulation for asymptomatic patients with incidentally detected HALT and preserved valve function. When HALT is accompanied by hemodynamically significant PVD, anticoagulation may be considered after individualized assessment of the overall clinical presentation, imaging findings, and potential thrombotic and bleeding risks [5,6,14]. The optimal therapeutic strategy and its effect on long-term valve durability remain uncertain.

HALT represents a dynamic imaging finding that may regress or newly develop during follow-up [2,3]. In the present case, the timing of HALT development cannot be determined because no cCT surveillance was performed between the early postoperative examination and the 12-month assessment. Therefore, this case does not establish early detection of HALT but instead documents the recognition of hemodynamically significant PVD associated with HALT and RLM at the time of clinical evaluation.

Although the optimal anticoagulation strategy after bioprosthetic valve replacement remains debated [15], this case highlights the importance of integrating clinical findings, valve hemodynamics, and multimodality imaging when evaluating suspected bioprosthetic valve dysfunction. Further studies are required to define the natural history of HALT after PerSV implantation and to determine which patients with leaflet thrombosis may benefit from anticoagulation therapy.

## Conclusions

A newly detected cardiac murmur may serve as a clinical trigger for further evaluation of prosthetic valve function, even in an asymptomatic patient. When unexpected hemodynamic prosthetic valve dysfunction is identified, cCT may help identify HALT and further characterize the underlying cause of valve dysfunction. In the present case, the resolution of HALT and reduced leaflet motion together with improvement in valve hemodynamics following anticoagulation supported a thrombotic mechanism, although further studies are needed to determine optimal surveillance and treatment strategies.
